# Adaptations to Swimming Training in Athletes with Down’s Syndrome

**DOI:** 10.3390/ijerph17249175

**Published:** 2020-12-08

**Authors:** José María González-Ravé, Anthony P. Turner, Shaun M. Phillips

**Affiliations:** 1 Sports Training Laboratory, Faculty of Sports Sciences, University of Castilla La Mancha, 45008 Toledo, Spain; 2 Sport, Physical Education & Health Sciences, University of Edinburgh, Edinburgh EH8 8AQ, UK; tony.turner@ed.ac.uk (A.P.T.); Shaun.Phillips@ed.ac.uk (S.M.P.)

**Keywords:** intellectual disabilities, strength, adapted sport, periodization

## Abstract

Swimming training programs may help to limit declines in cardiovascular conditioning, muscle strength, mobility and social functioning in individuals with Down’s Syndrome (DS): (1) Background: This study aims to analyze the effects of a periodized swimming training program on swimming speed, lower body force and power and body composition in a group of swimmers with DS; (2) Methods: Nine swimmers with DS (2 men and 7 women; aged 21–30 years-old) completed an 18-week periodized swimming program. The swimmers were assessed, pre and post-training, for 25 m, 50 m and 100 m freestyle swim performance, countermovement jump performance and body composition; (3) Results: Significant and large improvements in 25 m (mean −6.39%, *p* < 0.05, *d* = 1.51), 50 m (mean −4.95%, *p* < 0.01, *d* = 2.08) and 100 m (mean −3.08%, *p* < 0.05, *d* = 1.44) freestyle performance were observed following training, with no significant changes in body composition or consistent changes in jump performance (although a large mean 14.6% decrease in relative peak force, *p* < 0.05, *d* = 1.23) (4) Conclusions: A periodized 18-week training intervention may improve swimming performance in a small group of trained swimmers with DS, with less clear changes in jump performance or body composition. This program provides a training profile for coaches working with swimmers with DS and a platform for further research into the benefits of swimming training with this under-represented population.

## 1. Introduction

Down’s Syndrome (DS) is a genetic disorder caused by the presence of all or part of a third copy of chromosome 21, the most common chromosomal abnormality occurring in humans. Children with DS are typically limited by a variety of physical challenges including reduced strength, endurance, balance, neuromuscular control and cardio-respiratory fitness [1]. This contributes to individuals with DS being reported as less physically active than their peers without DS [2]. Furthermore, physical fitness in both youth and adults with DS is reduced in comparison to their peers without disabilities and those with other intellectual disabilities, but without DS [2]. So, effective policies and interventions to increase physical fitness in this specific population are urgently needed [3,4]. To improve the physical fitness of people with DS, numerous studies have focused on physiological adaptations such as increased cardiovascular fitness, muscle strength, and positive changes in body composition often seen after a training program [5,6,7]. Shields, Taylor and Dodd [8] investigated the effects of 10 weeks of resistance training (2 to 3 sets of 10–12 repetitions of 6 fixed weight exercises) in adults with DS. The intervention resulted in an increase in chest press endurance and also a trend toward an increase in upper-limb maximal strength as measured by a 1-RM test. Subsequently, Shields et al. [6] concluded that a 10-week supervised progressive resistance training program (twice a week using weight machines) appears to be an effective intervention to maintain physical activity levels in 68 young people with DS. A further study by Shields et al. [7] explored the association between physical activity, cardiovascular fitness and body size among children with DS, concluding that children with DS who had better cardiovascular fitness also had better body composition and lower waist circumference. While these physiological adaptations may improve performance of functional tasks [9], currently there is no research that investigates training to enhance sports performance in people with DS. Given evidence regarding improved strength following swimming training in adults without DS, and evidence that individuals with DS can show strength improvements, it is of interest to establish if swimming training in individuals with DS results in strength improvement.

There are few training studies in persons with DS in aquatic programs, especially longitudinally over a season. Boer and de Beer [5] propose that aquatic training may be an attractive alternative to land-based exercise for individuals with musculoskeletal conditions such as low muscle tone and excess adiposity, as found in adults with DS. The aforementioned study analyzed the effect of an aquatic training intervention (35 ± 10 min sessions, three times per week for 6 weeks) showing no significant changes in body mass, but significant improvements in aerobic capacity and a battery of functional tests related to strength, balance and endurance. Similar results were found in a recent study of Boer [3], but in specific tests showed contrasting results in 12 m and 24 m swimming time. In contrast, Ordonez et al. [4] demonstrated that a 12-week training intervention that included land and aquatic sessions showed a significant decrease in fat mass. Although the reported study precludes differentiating the effects of the aquatic and dryland training, and the participants were previously untrained, there is equivocal evidence regarding the effects of swimming training on body composition in adolescents with DS. Such uncertainty is also evident in a meta-analysis on adults with DS [10].

Such performance-oriented programs will typically adopt a model of periodization, the cyclic and gradual ordering of training exercises, following principles of specificity, volume and intensity to achieve high levels of sports performance at the most important competitions [11]. The aim is to enable progressive improvements on the specific qualities required for the athlete’s event via carefully balanced sport-specific strength and endurance training modalities [12]. From an inclusive perspective with DS athletes, training load prescription must be adapted to the physical characteristics and motor skills deficits aforementioned. Elite sprint British swimmers habitually completed, on average, 9.9 ± 0.3 swimming sessions per week [12]; Tate et al. [13] reported 7.7 ± 5.4 swimming sessions per week in U.S. college swimmers. However, to our knowledge there are no reported recommendations of volume of swimming sessions for swimmers with DS. In general, there remains a lack of information regarding the effectiveness of swim training on individuals with DS.

This study aimed to analyze the influence of a swimming training program on swimming performance, jump performance and body composition in swimmers with DS. It was hypothesized that swimming performance and jump performance would be improved with limited effect on body composition.

## 2. Materials and Methods

A single-group pretest-posttest design was adopted with a convenience sample, as we were unable to recruit a suitable matched control group (individuals with DS not enrolled in a swimming squad).

### 2.1. Participants

Nine swimmers with DS (2 men and 7 women; aged 21–30 years-old, height: 145 ± 6 cm) completed an 18-week periodized swimming program, from February to June. Based on Body Mass Index (BMI), the group were considered overweight at baseline (BMI 27.9 ± 6.0 kg/m^2^), with 3 participants with BMI > 30 kg/m^2^. All participants were members of a local swimming club who participate in adapted swimming competitions at regional and national level and are therefore considered trained but sub-elite. They were catalogued in the Federation of Intellectual Disabilities (which is the regional governing body of all sports, included swimming) as: G1 (six participants)—they were athletes whose motor skills allow them to participate in sport without modifications to the Regulations of the National Swimming Federation; and G2 (three participants)—those athletes whose motor skills need an adaptation of the Regulations of the National Swimming Federation. Baseline tests were performed in the week prior to the winter swimming regional competition in which the squad took part, and post-training tests were performed the week prior to the summer regional swimming competition (Table 1 and Table 2). All participants performed the same weekly training sessions on three days per week (Monday, Wednesday and Friday) with the same training volume and intensity. The daily workouts required a maximum of 90 min of training. The study was performed in accordance with the principles of the Declaration of Helsinki (October 2008, Seoul), and the experimental protocols were approved by the ethical committee of the local University (Approval Number FGM02102019). Parental consent was also obtained.

### 2.2. Training Intervention

A traditional linear periodization model was designed for the season, according to Bompa and Haff [10]. This approach aims to build aerobic capacity first through a period of high-volume/low-intensity training before reducing volume and increasing the proportion of high-intensity training [14]. The distribution of training intensity followed a polarized model [15], with training intensity expressed as a percentage of individual maximum heart rate (HRmax): Zone 1: 65–85% HRmax; Zone 2: 85–93% HRmax; Zone 3: 93–100% HRmax. We recorded HRmax as the highest HR achieved in the final 100 m of the incremental 8 × 100 m series adapted for swimmers with DS. Immediately upon completion of each swim HR was measured by the swimmer with a HR monitor. Unfortunately, we were unable to monitor training sessions continuously with HR monitors, as the club did not have access to enough monitors that functioned sufficiently accurately in the aquatic environment. It is recognized that there are challenges associated with using HRmax in swimmers with DS due to documented issues with chronotropic incompetence [16] we used a combination of %HR max and 100m swimming time to prescribe training zones and times for different series.

The season was divided into two mesocycles. The first mesocycle was conducted from week 23 to week 28, which involved the last part of the winter competition period (4 weeks) and the transition period (2 weeks). The baseline testing session and winter competition are shown in Table 1, as well as distribution of intensity and volume of training.

The second mesocycle of training was carried out over a period from week 29 to 42, divided into basic (build-up), specific, pre-competitive and competitive phases. Table 2 shows the post-intervention testing and the distribution of intensity and volume of training.

### 2.3. Procedure

Participants visited the facilities in a non-fatigued state (non-intense exercise in the previous 48 h). All testing sessions took place at the same hour of the day to avoid any influence of circadian rhythms and were led by the same researcher.

Tests were conducted in a randomized order on different dependent variables (body composition, countermovement jump (CMJ), and performance time in 25 m, 50 m and 100 m freestyle). Tests were performed on the same day in a biomechanics laboratory (body composition, countermovement jump) and an indoor 25 m swimming pool (25 m, 50 m and 100 m freestyle).

### 2.4. Swimming Performance

Before testing a standardized warm-up was completed: 200 m freestyle; followed by 100 m using legs only (alternating between 50 m of freestyle and 50 m of a different stroke); 100 m of arms only (alternating between 50 m of freestyle and 50 m of a different stroke); and, finally, 4 × 25 m freestyle with a progressive increase in speed. After the warm-up, the 25 m, 50 m, and 100 m freestyle sprint tests were performed with a recovery time of between 7 and 10 min. Participants were instructed to swim at their maximum speed. The timer was started manually when the swimmer’s feet left the starting block. The final time was measured by a timing plate (TP24, Alge Timing, Lustenau, Austria) that the swimmers touched at the end of the test.

### 2.5. Vertical Jump Test

The CMJ test was performed on a force plate (Quattro Jump 9290 DD, Kistler, Winterthur, Swiss) according to the protocol of Bosco, Luhtanen and Komi [17]. The participant stood in an upright position, flexed the knees and hips into a squat position (self-selected depth) and then immediately extended the knees and hips into an upward jump. The participants’ hands were kept on their hips during both jumps. Three maximal jumps were recorded with 30 s rest between attempts. Each participant was given external encouragement throughout all jumps. The attempt in which the highest jump height was obtained was used for analysis [18]. Jump height, mean force, relative peak force and mean power were calculated [17]. A possible limitation of the CMJ test in participants with DS is the difficulty in reproducing the correct technique. However, all participants in the current test understood the test instructions and procedures, and with no alternative aquatic-based assessment of lower-boy available, we elected to include the CMJ.

### 2.6. Body Composition

Body mass and height were measured using a balance scale to the nearest 0.1 kg (Seca, model 220, Hamburg, Germany) without shoes and clothes, and body composition by bioimpedance (Inbody 720, Biospace, Seoul, Korea), a valid tool for the assessment of total and segmental body composition [19]. To reduce measurement error, measurements were taken in the morning and at least 1 h after eating or drinking and by the same investigator.

### 2.7. Statistical Analysis

Data were checked for normality using the Shapiro–Wilk test before the analysis. Descriptive results are reported as means ± SD. Changes from baseline to post-intervention were also expressed as percentage change relative to the baseline scores and repeated measures t-tests were conducted to compare this percentage change compared to baseline (0%), accompanied by 95% Confidence Intervals for these differences. Cohen’s *d* was calculated as a measure of effect size (ES) and was interpreted as follows: small (<0.5), medium (<0.8) and large (≥0.8) [20].

## 3. Results

**Figure 1:**
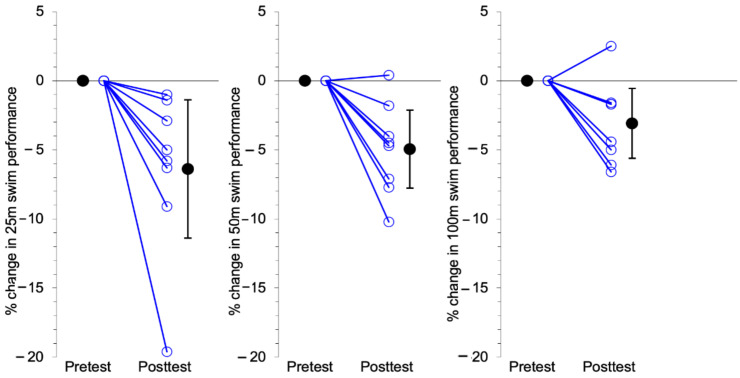
Individual (open blue circles) and mean (solid black circles with 95% Confidence Intervals) improvements in swimming performance times from pre- to post-training for 25 m, 50 m and 100 m freestyle sprints.

There were statistically significant improvements from baseline to post-training in swimming performance times for 25 m (6.39% mean improvement, *p* = 0.019, *d* = 1.51), 50 m (4.95% mean improvement, *p* = 0.004, *d* = 2.08) and 100 m (3.08% mean improvement. *p* = 0.024, *d* = 1.44) freestyle with large effect sizes (Table 3 and Figure 1).

In contrast, there were no statistically significant improvements in jump height, absolute mean force or mean power during the CMJ, although there was a statistically significant decrease in CMJ relative peak force with large effect size (14.6% mean decrease, *p* = 0.031, *d* = 1.23, Table 3). Finally, there were no statistically significant differences in markers of body composition, although a large effect size for increasing fat-free mass that was not statistically significant (Table 3). For both CMJ and body composition variables, there was a large amount of variation in the individual responses with large increases and decreases in some individuals, in contrast to the consistent swimming performance improvements.

## 4. Discussion

This is the first study to analyze the influence of a periodized swimming training intervention on swim performance, lower body force and power, and body composition in swimmers with DS. In support of the experimental hypotheses, there were large and statistically significant improvements in freestyle swimming performance over 25 m, 50 m and 100 m. In further support of the hypotheses, there were no statistically significant changes in body composition, however in contrast to the hypotheses there were no consistent changes in jumping performance, and even a statistically significant decrease in relative peak concentric force. Although lacking a control group comparison and uncertainty around the reliability of the CMJ, this study provides some initial pilot data on potential training improvements in an under-studied population.

The scientific literature has detailed the training programs of successful swimmers and squads [12,21,22], but there are no published studies focused on swimmers with DS. Session distance and intensity training data were obtained by direct observation of the training prescription indicated by coaches in their training schedule. The data reported here provide a single cohort example of a structured training program that can be used or adapted by other coaches planning their training over the competitive season in swimmers with DS, as well as some initial comparison data albeit with a small population.

There is a positive training effect with 25 m, 50 m and 100 m freestyle performance peaking just before the main competition scheduled in week 42, as intended with the periodized plan. The average training plan in the present study reflected principles of specificity more during taper than during build-up. The traditional training periodization is based on performing high-volume and low-intensity during the preparatory period (intervention weeks 6 to 13) and after that phase, the volume is slightly reduced and intensity is increased in weeks 17 and 18, [11,23]. Swimming coaches should consider increasing the low- and high-intensity training loads coinciding with the taper phase (from weeks 37 to 41) where an eventual drop of volume and an increase of intensity is planned, in order to obtain higher performance and avoid overtraining [21,22]. The volume of training carried out by swimmers with DS is lower than their peers without DS [13], even lower than daily volume recommended for 10-year-old swimmers [24]. These improvements were achieved through consistent training with a strong focus on intensities in zone 1 and a low volume of training in comparison to their peers without disabilities. In our study, the traditional linear periodization model followed a polarized model of distribution of intensity training, mainly in the competitive period, being efficient with these swimmers with this specific training program. These results support the positive association of performance progression and the gradually increasing of the workload for improvement of performance times according to the principle of progression of training. Our results showed that for 25 m performance time, one swimmer reported a 19% improvement, which was much greater than the other swimmers. This large improvement may in part be due to the shorter duration of the 25 m performance, but on closer inspection this swimmer was also initially the slowest and least trained of the group. Therefore, it is likely that this swimmer would respond more to almost any training and show the greatest improvements, both physiologically and in technique. In this sense Boer [3] showed an improvement in a 12 m swimming but not in 24 m due to the lack of specificity in training because participants never trained to swim 24 m. In this sense, the volume of training of our participants and the distribution of intensity training could support our results.

The lack of changes in body composition are in line with the results of Boer and de Beer [5] who performed an aquatic training intervention amongst adults with DS. This is possibly due to the low volume and intensity of the training sessions which was possibly not high enough during 18 weeks of training for decreasing body mass, although we presented a moderate to large effect size for positive improvements in fat-free mass which may indicate some positive adaptations. Furthermore, the diet of participants was neither controlled nor monitored, so it may well be that although physical activity increased, there may also have been an increase in dietary intake, offsetting any weight loss benefits. Shields et al. [6] showed that children with DS who had better cardiovascular fitness also had better body composition and lower waist circumference, and although we did not see a consistent and significant change, there were some indications of a slight decrease in body mass (−0.19%) and fat mass (−1.7%) as well as a moderate increase of fat free mass (2.4%). It is highly likely that large individual differences in body composition may mask a consistent effect within such a small sample size.

Potential contributing factors to the improvements in swim performance include improved strength and power, improved metabolic responses, cardio-respiratory adaptations [21,25,26], as well as changes to swimming technique and body shape (e.g., reduced drag). Based on the findings presented and the population used, it is unlikely that body shape changes will have contributed with no significant changes reported in body composition. Although CMJ performance did not improve, it remains possible that upper body strength and power were improved, which may contribute to improved performance, with only about 30 % of propulsion coming from the legs in freestyle [27]. In addition, we cannot differentiate between possible benefits of biomechanical changes and other physiological changes. Future studies exploring changes in technique (swimming efficiency and acyclic movements such as starts or turns) may elucidate the relative contributions of these mechanisms to the improved swimming performance.

The absence of dryland workouts oriented to strength and conditioning training may be a contributing factor to the lack of any demonstrable or statistically significant improvement in jump height, absolute mean force or mean power during the CMJ. Shields et al. [7] and Shields et al. [6] remarked on the positive contribution of resistance training on maximal strength and physical activity levels, but coaches have not included dryland workouts over this time, maybe due to the social and parental conditions of this specific population. The number of dryland workouts per week prescribed to elite swimmers for upper and lower body training appears to be an important factor which contribute to improved performance times in sprint distance swimmers (50–100) and 200 m in all strokes [26]. The demonstration of a statistically significant decrease in relative peak concentric force during the CMJ was surprising and not in line with the other CMJ variables. This may be the result of the difficulty that the participants had in performing the CMJ technique consistently, even within participants from baseline to post-training. There were very large standard deviations in CMJ performance and the CMJ values themselves are also of course very low. Therefore, we should be careful not to rule out potential lower body power benefits of the program in case movement variability and technique reduced the reliability of the results and potentially masked such benefits.

The results obtained in this study are limited to the specific participants and the low number of swimmers with DS in this squad. A control group who continued with their usual activities would have been desirable for the best control of the variables of the study, although some referenced trials were also limited because none employed a control group [3,6,7]—a common challenge in such research. Future studies with larger cohorts should be undertaken to confirm these results, including more balanced groups to explore any sex-specific differences in responses. The use of strength and conditioning sessions thorough the season would be recommended in order to detect any changes as a result of the strength training program (dryland workouts). 

In regard to body composition measures, significant discrepancies have been shown between DXA, so it could be considered that BIA is an inaccurate method for estimating body composition in individuals with DS. However, the results of Malavolti et al. [19] confirm that eight-polar BIA offers accurate estimates of total and appendicular body composition, besides, the reliability of BIA compared to other body composition measurement methods, like dual-emission X-ray absorptiometry (DXA), has been successfully demonstrated by others [28] and it was the only accessible method of body composition available within the feasibility constraints of this study.

This study adds to the body of knowledge as it is the first published evaluation of periodized swimming training in swimmers with DS. The data and presented periodized training program have practical relevance as a guide for the implementation of beneficial training practices in this population. From a health and fitness perspective, the meaningful positive responses in swimming performance to training promote swimming as a feasible exercise modality for improving physical activity levels and physical fitness in people with DS, both of which are lower than in other comparable populations. Increased physical activity and greater physical fitness associated with regular swimming participation may also improve more general health-related quality of life and provide psychosocial benefits associated with group activity participation. Future research that further investigates performance outcomes but also includes evaluation of specific physical, mental, and psychosocial health benefits from periodized swimming training would be useful additions to the literature. It is hoped this research will promote further studies in athletes with Down’s Syndrome, ideally employing study designs with larger sample sizes and control groups, to strengthen the evidence to support such interventions. However, this is recognized as a major challenge given the presence of neurological, biomechanical, and work capacity dysfunctions occurring simultaneously in individuals with DS.

## 5. Conclusions

In conclusion, performance times for swimming sprint distances of 25 m, 50 m and 100 m were improved in a small population of trained swimmers with DS, following an 18-week traditional linear periodized training program with a polarized distribution of intensity training and low volume (ranged between 1000 to 1800 m per daily session). These exploratory data support the potential use of swim training in this population, despite no substantial changes in body composition and forces produced in dry land conditions, which require further controlled research.

## Figures and Tables

**Table 1:** Characteristics of competitive and transition period in Macrocycle 1.

Month	February		March			
Competition	Baseline Test			C		
Period	Competitive				Transition	
Intervention week	0	1	2	3	4	5
Week of season	23	24	25	26	27	28
DTI (% of total training time)	z1: 87.2 ± 1.2% z2: 6.1 ± 0.7% z3: 6.6 ± 0.9%				z1: 96.3 ± 1.1% z2: 2.2 ± 0.7% z3: 1.5 ± 0.9%	
Training days/week	3	3	3	3	2	2
Daily volume (m)	1600	1000	1100	1000	1500	1800
Session duration (h)	1.5	1	1.5	1	1.5	1.5

Note. DTI: Distribution of Training Intensity (z1, z2, z3: zones 1–3 were 65–85%, 85–93% and 93–100% of maximum heart rate respectively). C denotes competition.

**Table 2:** Macrocycle 2 characteristics.

Month	March	April				May					June			
Competition			C		C							C	Post-Test	Main C
Period	Preparation (Basic)				Preparation (Specific)				Pre-Competitive			Competitive		
Intervention week	6	7	8	9	10	11	12	13	14	15	16	17	18	19
Week of season	29	30	31	32	33	34	35	36	37	38	39	40	41	42
DTI (% of total training time)	z1: 90.2 ± 2.2% z2: 6.1 ± 1% z3: 4.3 ± 1%				z1:86.2 ± 2.1% z2: 6.8 ± 0.7% z3: 6.6 ± 0.9%				z1: 82.2 ± 2% z2: 1 ± 2% z3: 8 ± 1%					
Training days/week	3	2	3	3	3	2	3	3	3	3	3	3	3	3
Daily volume (m)	1500	1400	1200	1800	1600	1700	1400	1400	1400	1500	1600	1000	1100	1000
Session duration (h)	1.5	1.2	1.5	1.5	1.5	1.5	1.5	1.5	1.2	1.2	1.2	1	1.2	1

Note. DTI: Distribution of Training Intensity (z1, z2, z3: zones 1–3 were 65–85%, 85–93% and 93–100% of maximum heart rate respectively). C denotes competition.

**Table 3:** Mean (SD) values for swimming performance, jump performance and body composition pre- and post-training, and the mean relative change (%) following the 18-week swimming intervention.

Dependent Variables	Baseline	Post−Training	Mean % Change	*p*−Value	Cohen’s *d*
25m freestyle time (s)	29.75 (10.8)	27.35 (7.9)	−6.39 *	0.019	1.51
50 m freestyle time (s)	62.55 (16.49)	59.5 (16.06)	−4.95 *	0.004	2.08
100 m freestyle time (s)	139.48 (57.36)	135.78 (57.2)	−3.08 *	0.024	1.44
CMJ jump height (cm)	0.14 (0.03)	0.15 (0.02)	8.4	0.157	0.74
CMJ relative peak force (%BW)	166.2 (29.5)	139.3 (24.3)	−14.6 *	0.031	1.23
CMJ mean force (N)	835.4 (208.1)	818.8 (250.6)	−2.28	0.558	0.29
CMJ mean power (W)	889.9 (100.1)	855.8 (230.4)	3.9	0.635	0.23
Body Mass (kg)	58.3 (12.2)	57.9 (11.4)	−0.19	0.909	0.06
Fat Mass (kg)	21.7 (10.5)	21.01 (10.4)	−1.07	0.825	0.11
Fat−Free Mass (kg)	19.6 (3.7)	20.0 (3.5)	2.4	0.144	0.76

Note. * statistically significant change, *p* < 0.05.
